# The incidence of gingival recession with non-surgical crossbite correction using completely customized lingual appliances versus surgically assisted rapid palatal expansion in adults: a cohort study

**DOI:** 10.1186/s40510-025-00568-0

**Published:** 2025-07-01

**Authors:** Jonas Q. Schmid, Lara Bettenhäuser-Hartung, Moritz Kanemeier, Ariane Hohoff, Johannes Kleinheinz, Thomas Stamm, Claudius Middelberg, Yann Janssens

**Affiliations:** 1 University of Münster, Münster, Germany; 2 Hannover Medical School (MHH), Hannover, Germany; 3 Private Practice, Bad Essen, Germany; 4 Université Paris Cité, Paris, France

**Keywords:** Crossbite, Surgically assisted rapid palatal expansion, Surgically assisted rapid maxillary expansion, Dentoalveolar compensation, Expansion, Mandibular constriction, Mandibular compression, Lingual orthodontics, Gingival recession, Soft tissue

## Abstract

**Background:**

The aim of this study was to investigate if there is a significant difference in the incidence of buccal gingival recession after non-surgical transversal dentoalveolar compensation with completely customized lingual appliances (DC-CCLA) versus surgically assisted rapid palatal expansion (SARPE).

**Methods:**

This cohort study included 81 adult patients with posterior crossbite. The DC-CCLA group (n = 38; f/m 25/13; mean age 30.3 ± 13.0 years) was treated with dentoalveolar compensation using completely customized lingual appliances. The SARPE-group (n = 43; f/m 19/24; mean age 28.2 ± 9.4 years) was treated with SARPE and buccal straight wire appliances. The number of buccal gingival recessions was recorded on digital models before treatment (T0) and after removal of fixed appliances (T1). Statistical analyses included Fisher’s exact tests, Chi-squared tests, Mann-Whitney U tests and mixed-effects logistic regression to evaluate the influence of various variables on the incidence of gingival recession.

**Results:**

In 3976 teeth evaluated, the incidence of developing gingival recessions was 8.1% with DC-CCLA (n = 77) and 5.8% with SARPE (n = 60). This difference was not statistically significant (p > 0.05). Age was a significant factor for the incidence of gingival recession and recessions were more likely to occur in males and in the maxillary posterior region.

**Conclusions:**

There was no statistically significant difference in the incidence of gingival recessions between dentoalveolar compensation with CCLAs and SARPE after debonding, with some degree of gingival recession being inevitable with both treatment approaches.

## Introduction

Posterior crossbite is a common malocclusion with a prevalence of up to 15% in the European population [1]. Treatment options for posterior crossbite in adults commonly include surgically assisted rapid palatal expansion (SARPE), segmental osteotomies, microimplant-assisted rapid palatal expansion (MARPE) or dentoalveolar compensation. Each option has certain advantages and disadvantages.

SARPE is a surgical procedure for transversal widening of the maxilla in skeletally mature patients, with the aim of weakening the main resistance to expansion. To date, there is no consensus about the indications for SARPE [2, 3]. While the theory of bone-borne SARPE - less dental side effects and smaller relapse - seems promising [4], studies have failed to show significant differences for skeletal/dental effects and relapse with bone-borne or tooth-borne SARPE [5–8]. According to expert opinion, transverse maxillomandibular discrepancies of up to 5 mm can be corrected non-surgically by dentoalveolar compensation [9]. However, a retrospective study has shown that even larger amounts of transverse discrepancies can be corrected by dentoalveolar compensation using completely customized lingual appliances (CCLAs) when the correction is performed in both jaws using expansion archwires in the upper jaw and compression archwires in the lower jaw [10]. A recent systematic review of randomized controlled trials found that SARPE has less skeletal effects than commonly expected and can be considered primarily as a molar expansion procedure [11]. As the skeletal effects of SARPE are small and complications such as severe bleeding, postoperative pain, inadequate/asymmetrical expansion are common [12, 13], this draws attention to dentoalveolar compensation as one possible therapeutic option for adults with posterior crossbite. When transversal dentoalveolar compensation is performed, questions about tipping of posterior teeth exist. It has been shown that the buccolingual inclination change is comparable between SARPE and dentoalveolar compensation with CCLAs [14]. There are also questions about the development of gingival recession. It was shown that adolescent patients with a crossbite before treatment showed more new recessions than patients without a transverse maxillomandibular discrepancy [15]. To date, there is a lack of evidence whether posterior crossbite correction leads to gingival recession in adults.

It is well known that mucogingival conditions can be adversely affected by orthodontic tooth movements [16, 17]. According to the current Classification of Periodontal Diseases and Conditions, gingival recession is defined as an apical shift of the gingival margin caused by different conditions/pathologies [18]. Gingival recession is common in adults, occurs in orthodontically untreated patients, and the prevalence increases with age [19, 20]. Thin gingival phenotype is considered a risk factor for the development of gingival recession [18, 21, 22]. Gingival recession can lead to undesirable consequences, such as impaired aesthetics, dentin hypersensitivity, cervical caries, and non-carious cervical lesions [18]. The prevalence of gingival recession immediately after orthodontic treatment is 5–7% and increases significantly in the post-treatment period [23, 24], with no significant difference from orthodontically untreated patients after 10–15 years [15]. However, it remains unclear which tooth movements lead to gingival recession [25–27], but it has been shown that orthodontic torque correction can reduce gingival recession depth [28]. Transverse dentoalveolar compensation is often considered to have a greater risk of gingival recession. However, there is a lack of data on this topic.

The aim of this study was to compare the number of gingival recessions after non-surgical transversal dentoalveolar compensation with completely customized lingual appliances (DC-CCLA) versus SARPE. The null hypothesis was tested that there is no significant difference in the number of gingival recessions between patients treated with DC-CCLA and patients treated with SARPE after active orthodontic treatment.

## Materials and methods

This cohort study is a follow-up study on DC-CCLA compared to SARPE. The same patients were included as in the two previous investigations [10, 14]. The study protocol was approved by the local Ethics Commission of the Medical Faculty of the University of Münster, Germany (2021-120-f-S) and the study was reported according to the STROBE Guidelines [29]. Measurements were performed at the University Hospital Münster, Germany. Two groups were formed to compare gingival recession: The surgical group was treated with SARPE followed by a buccal straight wire appliance. The non-surgical DC-CCLA group was treated with a CCLA (WIN, DW-Lingual Systems GmbH, Bad Essen, Germany). Gingival recessions were recorded on digital models at two time points: before treatment (T0) and after removal of fixed appliances (T1). The inclusion criteria were: (1) adult patients over the age of 18, (2) presence of a posterior crossbite involving at least two teeth per jaw, (3) Angle Class I, II, or III occlusion, and (4) availability of models at (T0) and (T1). Exclusion criteria were: (1) patients with orofacial syndromes, (2) patients with cleft conditions, (3) presence of tooth agenesis, (4) presence of periodontal disease requiring periodontic treatment, and (5) teeth that were moved in the sagittal direction for space opening or closure.

**Fig. 1:**
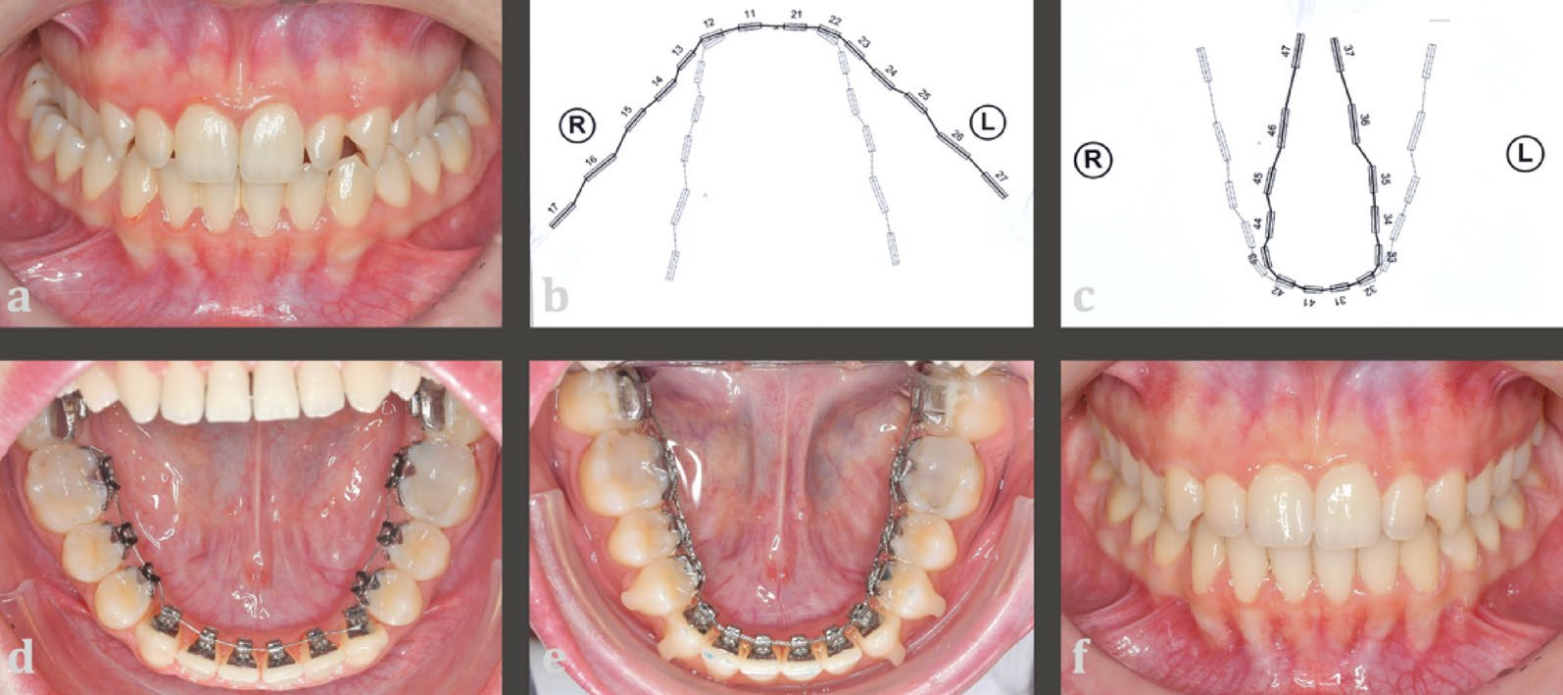
Clinical procedure in the non-surgical DC-CCLA group. **a** Pre-treatment situation with bilateral crossbite. **b** Expansion archwire in the upper jaw. **c** Compression archwire in the lower jaw. **d** Levelling and aligning with a completely customized lingual appliance. **e** Situation after compression in the lower jaw. **f** Post-treatment situation after non-surgical crossbite correction

Consecutively treated adult patients from a private practice (Bad Essen, Germany) whose fixed lingual appliances were removed between 2019 and 2021 were eligible for inclusion in the DC-CCLA group. Individual 0.016 × 0.024-inch stainless steel CAD/CAM archwires with up to 3 centimetres of expansion in the upper jaw and compression archwires with up to 2 centimetres compression in the lower jaw were used (Fig. 1) [30].

**Fig. 2:**
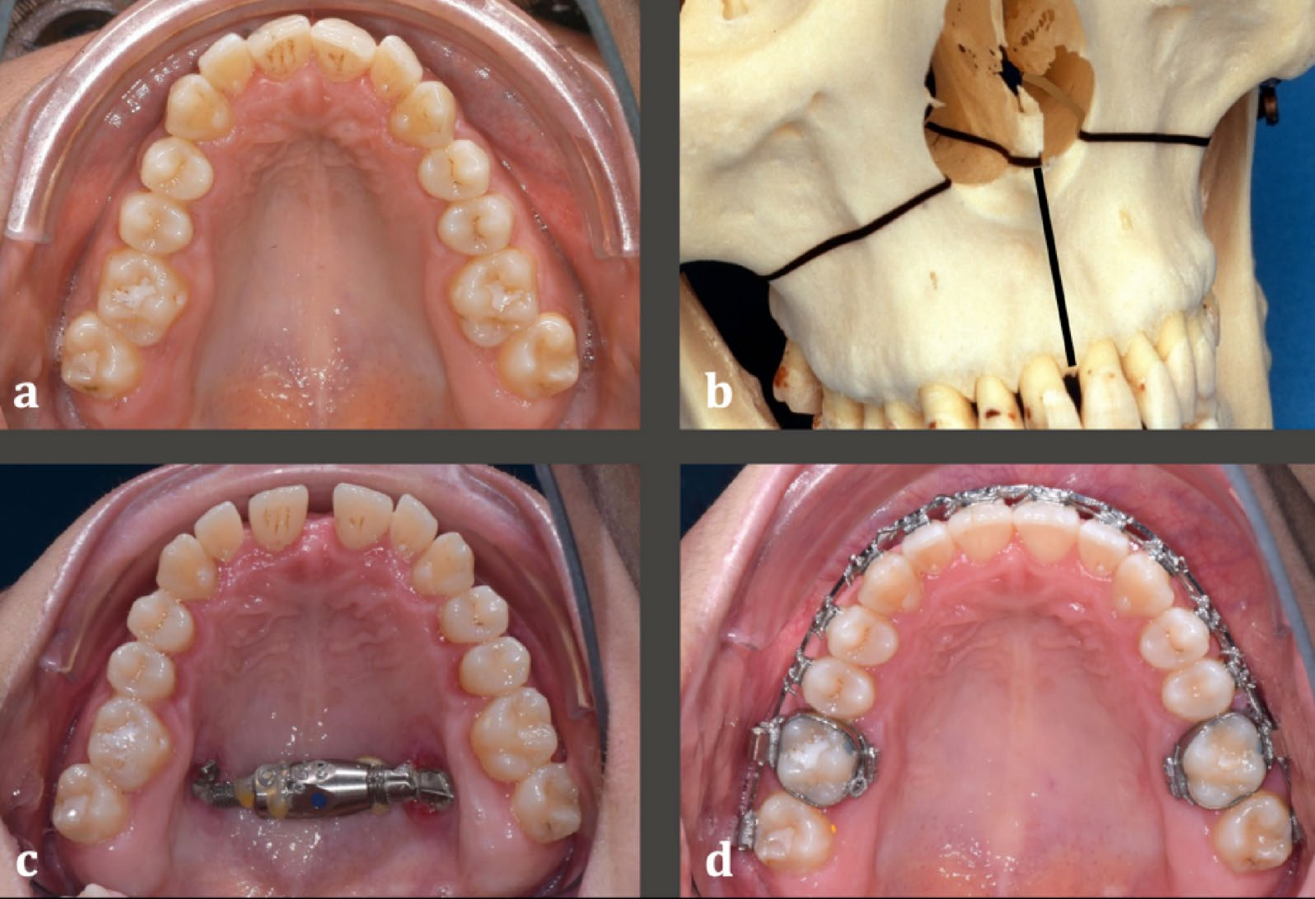
Clinical procedure in the surgical SARPE group. **a** Pre-treatment situation with narrow upper jaw. **b** The surgical procedure involved a subtotal Le-Fort I osteotomy with separation of the pterygomaxillary junction. **c** Bone-borne expansion appliance after activation. **d** Buccal straight wire appliance for levelling and alignment prior to single- or two-jaw surgery

Eligible for inclusion in the SARPE group were adult patients who consecutively underwent surgery at the Department of Cranio-Maxillofacial Surgery, University Hospital Münster, Germany from 2018 to 2021. All patients were treated with subtotal Le-Fort I osteotomy with separation of the pterygomaxillary junction and buccal straight wire appliances (Fig. 2). Bone-borne or tooth-borne expansion appliances were used, and all patients received buccal straight wire appliances for levelling and alignment. Every patient subsequently received orthognathic surgery with a bilateral sagittal split osteotomy (BSSO) or a BSSO in combination with a Le-Fort I osteotomy. A marginal incision was performed for the surgical interventions as previously described [31].The orthognathic surgeries were planned with the Digital Münster Model Surgery (DMMS) system [32, 33].

The number of consecutively treated patients in the study period determined the sample size in both groups.

### Registration of gingival recession

**Fig. 3:**
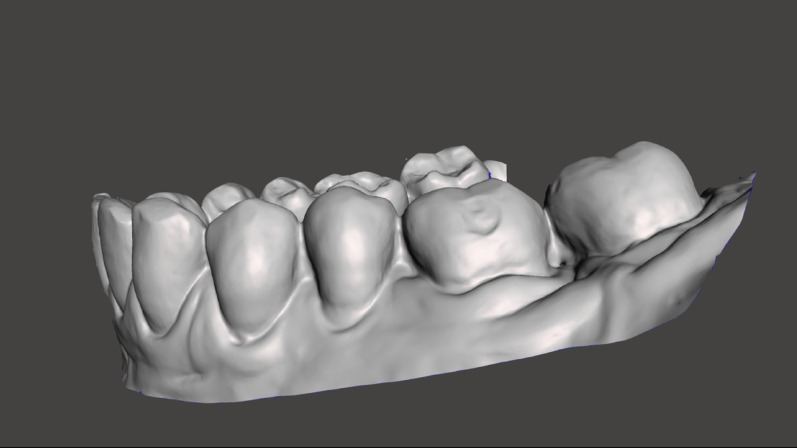
Registration of buccal gingival recession on digital models before treatment (T0) and after removal of fixed appliances (T1). The model shown is from T1

Primary outcome was the presence of a labial gingival recession on each tooth (Yes/No) at T0 and T1, similar to the methodology of Renkema et al. [23]. Two experienced orthodontists meticulously evaluated the digital models together using a consensus-based approach, acting as one single examiner. Prior to scoring, both raters underwent calibration to minimize variability. A consensus was reached if the scoring between the raters differed. A gingival recession was defined according to the consensus report of workgroup 3 of the 2017 World Workshop on the Classification of periodontal and peri-implant diseases and conditions as an apical shift of the gingival margin [18]. A recession was scored if the labial root surface was clearly exposed (Fig. 3). Teeth were classified as valid if registration of gingival recession was possible at the T0 and T1 model. Teeth that could not be evaluated at T0 and/or T1, teeth that were moved in the sagittal direction, or missing teeth were classified as non-valid. The raters were blinded to the treatment groups, as all digital models were assigned pseudonyms that masked the identity of the group.

### Statistical analysis

Intrarater reliability for the detection of gingival recession was evaluated using Cohen’s Kappa. Level of agreement was interpreted according to Landis and Koch [34]. For this purpose, 10% of the sample (8 patients) were selected using a random numbers generator and rescored after at least four weeks by the same experienced orthodontists using the consensus-based approach. Descriptive statistics were calculated for all variables. Chi-squared tests and Mann-Whitney U tests were used to evaluate differences in the baseline characteristics. Fisher’s exact tests, Chi-squared tests and Mann-Whitney U tests were used to assess intergroup differences. Non-parametric tests were used since the data was not normally distributed, as assessed by the Shapiro-Wilk test (*p* < 0.05). Alpha correction for multiple comparisons was performed using the Benjamini-Hochberg procedure.

Mixed-effects logistic regression with forward variable selection based on likelihood ratio tests was performed to evaluate the influence of gender, age at T0, observation time, Angle class (I, II, III), expansion appliance (bone-borne, tooth-borne, DC-CCLA), tooth region (maxillary anterior, maxillary posterior, mandibular anterior, mandibular posterior) and intervention (SARPE, DC-CCLA) on the incidence of gingival recession.

The significance level was set at 5%, with p-values less than 0.05 considered significant. All statistical analyses were performed using R (version 4.3.2) [35]. Mixed-effects logistic regression was performed using the lme4 package [36] and confidence intervals for the estimates were calculated using lmerTest [37].

## Results

81 patients met the inclusion criteria. The baseline characteristics are shown in Table 1. Cohen’s Kappa was used to evaluate intrarater reliability for the detection of gingival recession. There was almost perfect agreement between the two time points (κ = 0.876, *p* < 0.001). The mean observation time (T1-T0) was 3.8 ± 1.6 years in the SARPE group and 2.5 ± 1.1 years in the DC-CCLA group. A total of 24 patients (55.8%) in the SARPE group received tooth-borne expansion appliances and 19 patients (44.2%) bone-borne appliances (Table 1).

**Table 1:** Baseline characteristics and intergroup Chi-squared and Mann-Whitney U tests

	SARPE	DC-CCLA	p
Age T0 (years) Mean ± SD	28.1 ± 9.4	30.3 ± 13.0	0.818
Age T1 (years) Mean ± SD	31.9 ± 9.7	32.9 ± 13.1	0.712
Observation T1-T0 (years) Mean ± SD	3.8 ± 1.6	2.5 ± 1.1	< 0.001
Gender n (%)			0.051
Female	19 (44.2%)	25 (65.8%)	
Male	24 (55.8%)	13 (34.2%)	
Angle Class n (%)			< 0.001
Class I	2 (4.7%)	20 (52.6%)	
Class II	16 (37.2%)	13 (34.2%)	
Class III	25 (58.1%)	5 (13.2%)	
Expansion appliance n (%)			
Tooth-borne	24 (55.8%)		
Bone-Borne	19 (44.2%)		

A total of 3976 valid teeth were evaluated. Frequencies for the number of gingival recessions before treatment (T0), after treatment (T1), the difference in the number of gingival recessions (T1-T0), and intergroup test statistics are shown in Tables 2, 3 and 4.

**Table 2:** Frequencies of gingival recessions before treatment (T0) and intergroup test statistics

Localisation	SARPE		DC-CCLA		Intergroup differences (p values)
	n valid	n rec	n valid	n rec	
17	37	1	34	1	1.000
16	35	4	37	9	1.000
15	37	5	37	6	1.000
14	36	5	30	8	1.000
13	39	2	34	5	1.000
12	39	5	36	1	1.000
11	39	1	35	2	1.000
21	39	1	36	5	1.000
22	36	2	34	4	1.000
23	38	7	30	4	1.000
24	34	8	32	6	1.000
25	35	2	33	4	1.000
26	34	5	35	6	1.000
27	39	0	33	2	1.000
Maxilla n/%	517/85.9%	48/9.3%	476/89.5%	63/13.2%	0.097
37	36	0	37	0	1.000
36	31	5	31	3	1.000
35	38	4	29	4	1.000
34	37	7	32	7	1.000
33	42	3	35	2	1.000
32	39	2	35	4	1.000
31	36	1	36	4	1.000
41	39	1	36	2	1.000
42	41	2	37	2	1.000
43	41	2	34	4	1.000
44	35	4	34	6	1.000
45	34	6	31	5	1.000
46	34	2	33	1	1.000
47	36	0	36	1	1.000
Mandible n/%	519/86.2%	39/7.5%	476/89.5%	45/9.5%	0.272
Total n/%	1036/86.0%	87/8.4%	952/89.5%	108/11.3%	0.027

**Table 3:** Frequencies of gingival recessions after treatment (T1) and intergroup test statistics

Localisation	SARPE		DC-CCLA		Intergroup differences (p values)
	n valid	n rec	n valid	n rec	
17	37	2	34	3	0.916
16	35	2	37	14	0.038
15	37	4	37	9	0.772
14	36	8	30	11	0.772
13	39	6	34	3	0.916
12	39	3	36	2	1.000
11	39	5	35	3	0.916
21	39	7	36	4	0.916
22	36	3	34	4	0.916
23	38	7	30	5	1.000
24	34	12	32	14	0.916
25	35	3	33	4	0.916
26	34	4	35	14	0.175
27	39	0	33	3	0.754
Maxilla n/%	517/85.9%	66/12.8%	476/89.5%	93/19.5%	0.007
37	36	0	37	3	0.772
36	31	7	31	6	1.000
35	38	4	29	8	0.754
34	37	7	32	10	0.772
33	42	3	35	4	0.916
32	39	2	35	3	0.916
31	36	3	36	5	0.916
41	39	4	36	4	1.000
42	41	1	37	4	0.772
43	41	1	34	4	0.772
44	35	7	34	5	0.916
45	34	6	31	3	0.916
46	34	5	33	6	0.916
47	36	1	36	2	1.000
Mandible n/%	519/86.2%	51/9.8%	476/89.5%	67/14.1%	0.038
Total n/%	1036/86.0%	117/11.3%	952/89.5%	160/16.8%	< 0.001

**Table 4:** Difference in the number of gingival recessions (T1-T0) and intergroup test statistics

Localisation	SARPE				DC-CCLA				intergroup differences (p values)		
	n valid	n rec	n rec +	n rec −	n valid	n rec	n rec +	n rec −	n rec^†^	n rec +	n rec −
17	37	1	1	0	34	2	3	1	0.809	0.846	1.000
16	35	−2	0	2	37	5	7	2	0.245	0.324	1.000
15	37	−1	2	3	37	3	5	2	0.569	0.846	1.000
14	36	3	4	1	30	3	5	2	0.879	1.000	1.000
13	39	4	4	0	34	−2	0	2	0.161	0.662	1.000
12	39	−2	1	3	36	1	1	0	0.566	1.000	1.000
11	39	4	4	0	35	1	1	0	0.566	0.846	1.000
21	39	6	6	0	36	−1	0	1	0.161	0.362	1.000
22	36	1	1	0	34	0	1	1	0.809	1.000	1.000
23	38	0	3	3	30	1	2	1	0.809	1.000	1.000
24	34	4	5	1	32	8	8	0	0.566	0.846	1.000
25	35	1	1	0	33	0	0	0	0.647	1.000	1.000
26	34	−1	2	3	35	8	8	0	0.161	0.640	1.000
27	39	0	0	0	33	1	3	2	0.809	0.640	1.000
Maxilla n/%	517/85.9%	18/3.5%	34/6.6%	16/3.1%	476/89.5%	30/6.3%	44/9.2%	14/2.9%	0.205	0.199	0.888
37	36	0	0	0	37	3	3	0	0.482	0.846	1.000
36	31	2	4	2	31	3	4	1	0.843	1.000	1.000
35	38	0	2	2	29	4	4	0	0.503	0.846	1.000
34	37	0	2	2	32	3	4	1	0.569	0.846	1.000
33	42	0	1	1	35	2	2	0	0.569	0.935	1.000
32	39	0	2	2	35	-1	0	1	0.809	0.866	1.000
31	36	2	2	0	36	1	2	1	0.809	1.000	1.000
41	39	3	3	0	36	2	2	0	0.809	1.000	1.000
42	41	−1	1	2	37	2	2	0	0.566	0.935	1.000
43	41	−1	0	1	34	0	1	1	0.809	0.846	1.000
44	35	3	4	1	34	−1	1	2	0.566	0.846	1.000
45	34	0	1	1	31	−2	1	3	0.691	1.000	1.000
46	34	3	3	0	33	5	6	1	0.756	0.846	1.000
47	36	1	1	0	36	1	1	0	1.000	1.000	1.000
Mandible n/%	519/86.2%	12/2.3%	26/5.0%	14/2.6%	476/89.5%	22/4.6%	33/6.9%	11/2.3%	0.205	0.199	0.888
Total n/%	1036/86.0%	30/2.9%	60/5.8%	30/2.9%	952/89.5%	52/5.5%	77/8.1%	25/2.6%	0.061	0.051	0.785

*n rec + * number of teeth where gingival recession developed (T1-T0),* n rec −* number of teeth where gingival recession disappeared (T1-T0)

† Mann-Whitney U test statistics

### Number of gingival recessions before treatment (T0)

In the investigated adult population, multiple gingival recessions were already present before treatment (T0). Overall, 87 teeth in the SARPE group and 108 teeth in the DC-CCLA group were affected by gingival recessions at T0 (Table 2). The tooth related prevalence of gingival recessions at T0 was 8.4% in the SARPE group and 11.3% in the DC-CCLA group and in total, this difference was statistically significant (Table 2). Recessions were unevenly distributed within the dental arches, with some teeth more frequently affected. In the SARPE group, maxillary left first premolars were most often affected followed by mandibular left first premolars, and mandibular second molars were the least frequently affected teeth. In the DC-CCLA group, maxillary right first molars were most often affected followed by maxillary right first premolars, and mandibular left second molars were the least commonly affected teeth. However, there was no statistically significant difference in the number of gingival recessions at time T0 between the groups for individual teeth (Table 2).

### Number of gingival recessions after treatment (T1)

There was an increase in the number of gingival recessions after treatment compared to before treatment. Overall, 117 teeth in the SARPE group and 160 teeth in the DC-CCLA group had gingival recessions at T1 (Table 3). The tooth related total prevalence of gingival recessions at T1 was 11.3% in the SARPE group and 16.8% in the DC-CCLA group. Similar to T0, there was a statistically significant difference in the number of gingival recessions at time T1 between the groups (Table 3). The distribution of gingival recessions within the arches was similar to T0: In the SARPE group, maxillary left first premolars again were most often affected followed by mandibular left first premolars, and mandibular left second molars were the least frequently affected teeth. In the DC-CCLA group, both maxillary first molars and the maxillary left first premolar were most often affected, followed by the maxillary right first premolar. For individual teeth, there was a statistically significant difference in the number of gingival recessions at T1 between the groups only for tooth 16 (Table 3).

### Difference in the number of gingival recessions (T1-T0)

**Fig. 4:**
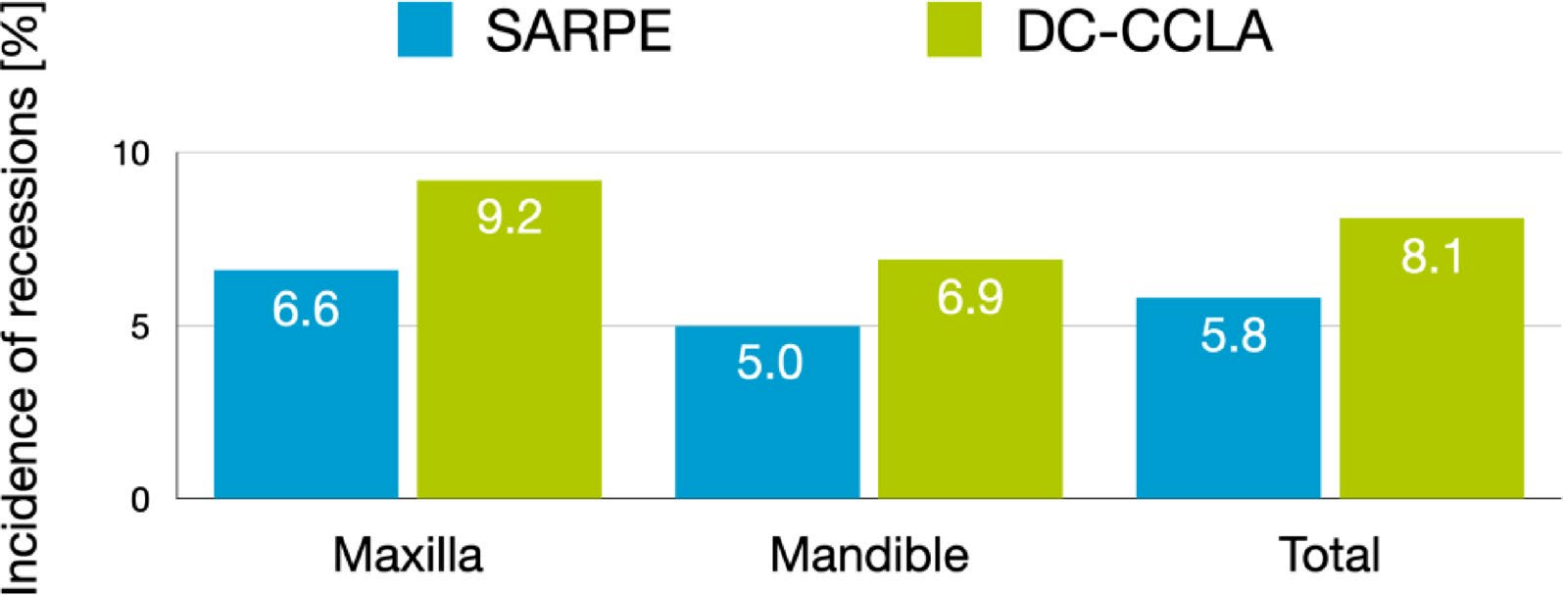
Tooth related incidence of developing a buccal gingival recession in the SARPE group and the DC-CCLA group

**Fig. 5:**
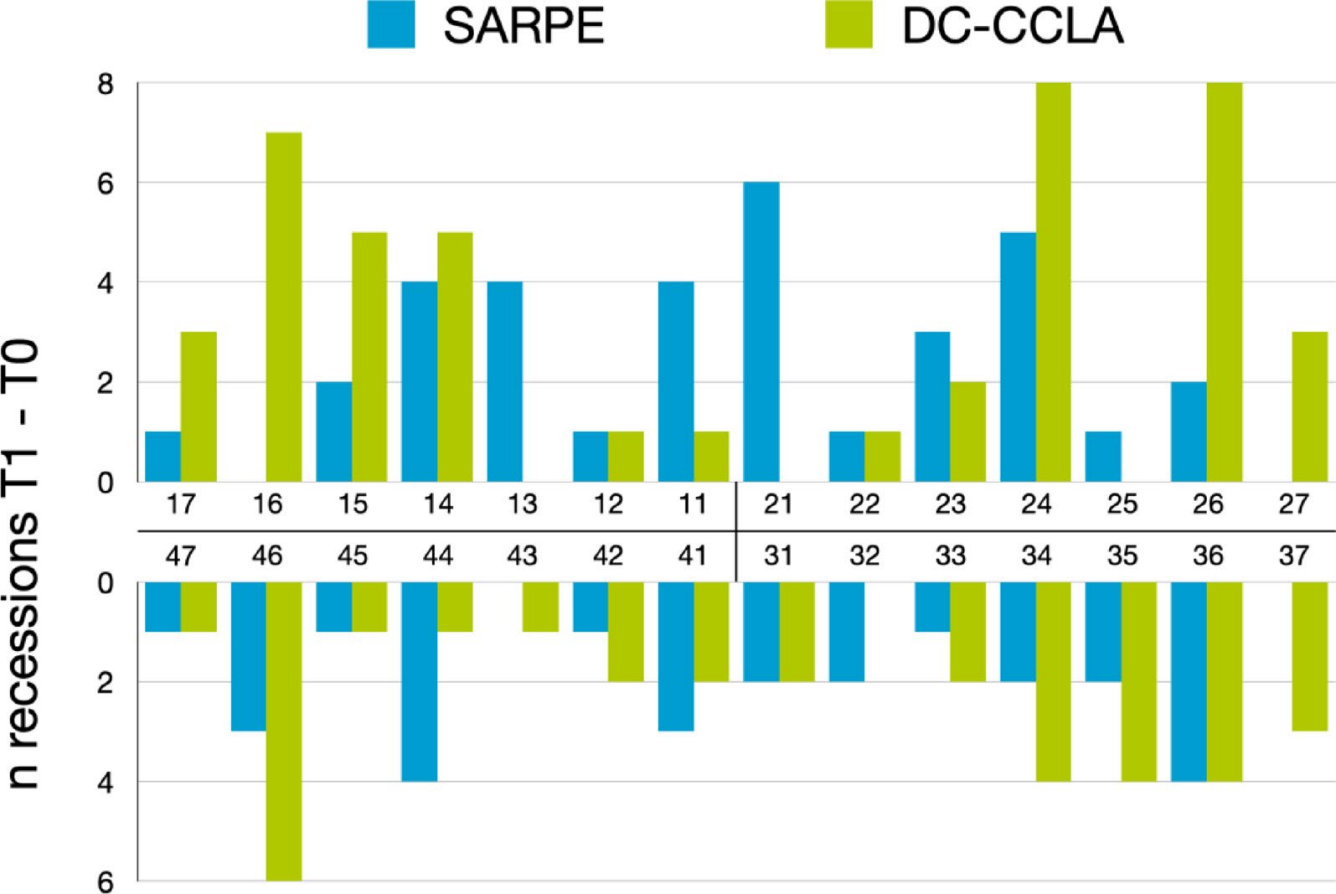
Number of teeth that developed a gingival recession in the SARPE group and the DC-CCLA group

In summary, 60 teeth in the SARPE group and 77 teeth in the DC-CCLA group that showed no gingival recession at T0 developed a gingival recession (Table 4). The total tooth related incidence of developing a gingival recession was 5.8% with SARPE and 8.1% with DC-CCLA (Fig. 4). This difference was not statistically significant. Notably, during treatment, gingival recession disappeared at 30 teeth in the SARPE group and 25 teeth in the DC-CCLA group - these teeth had gingival recession at T0 and no gingival recession at T1. Some teeth were more frequently affected by developing a gingival recession (Table 4; Fig. 5). In the SARPE group, from T0 to T1 maxillary teeth were most often affected by gingival recessions: Left central incisor (+ 6), left first premolar (+ 5), right central incisor, right first canine and right first premolar (+ 4 each). Maxillary/mandibular left second molars, maxillary right first molars as well as mandibular right canines were not affected by gingival recessions from T0 to T1. In a subgroup analysis, the influence of the expansion appliance was examined: There was no statistically significant difference (*p* > 0.05) in the incidence of gingival recession with either tooth or bone-borne appliances in the maxilla and for maxillary teeth. In the DC-CCLA group, maxillary left first molars and left first premolars (+ 8 each), right first molars (+ 7) as well as right first and second premolars (+ 5 each) were most frequently affected by gingival recessions from T0 to T1. Maxillary left central incisors were the least frequently affected teeth. However, intergroup differences for individual teeth were not statistically significant.

### Mixed-effects logistic regression

To assess the influence of different variables on the incidence of gingival recession a mixed-effects logistic regression was performed. In the forward selection, age (χ² (1) = 10.68, *p* = 0.001), gender (χ² (1) = 4.26, *p* = 0.039), tooth region (χ² (3) = 9.95, *p* = 0.019) and appliance (χ² (2) = 6.68, *p* = 0.04) were found to significantly improve the null model, while observation time (χ² (1) = 0.17, *p* = 0.679), Angle class (χ² (2) = 0.07, *p* = 0.966) and intervention (χ² (1) = 2.35, *p* = 0.125) did not. Following the forward selection, appliance (χ² (2) = 5.25, *p* = 0.07) was excluded. The final model included the predictors age, gender, and tooth region.

The estimated log odds for each predictor are shown in Table 5. Age, male gender, and the maxillary posterior tooth region were positively associated with the incidence of gingival recession. The incidence of gingival recession was independent of the type of intervention performed. The estimated log odds correspond to odds ratios of 1.04 for age, 2.02 for male gender, and 2.13 for the maxillary posterior tooth region. The odds of gingival recession increased by 4.2% for each additional year of age, were 102.4% higher in males and 113.0% higher in the maxillary posterior tooth region compared to the mandibular anterior tooth region. In summary, age was a significant factor in the development of gingival recession and recession was more likely to occur in males and in the maxillary posterior region.

**Table 5:** Estimated log-odds of the mixed-effects logistic regression for the incidence of gingival recession

Predictor	Estimated log (OR)	95% CI		p
Intercept	-4.890	-5.954	-3.953	< 0.001
Age	0.041	0.020	0.064	< 0.001
Gender (male)	0.705	0.197	1.249	0.007
Tooth region (maxillary anterior)	0.064	-0.703	0.836	0.870
Tooth region (maxillary posterior)	0.756	0.173	1.404	0.015
Tooth region (mandibular posterior)	0.378	-0.227	1.042	0.239

## Discussion

This study compared the effects of SARPE and DC-CCLA on gingival recession in adults with posterior crossbite. It was found that both treatments led to an increase in gingival recession after debonding. There were no statistically significant overall differences between the groups, and gingival recession was observed regardless of the type of intervention that was performed. The odds for developing gingival recession were higher in males, in the maxillary posterior region, and with increasing age. These findings are consistent with the existing literature, showing that orthodontic treatment in general can lead to gingival recession [16]. The null hypothesis was accepted. There was no statistically significant difference in the number of gingival recessions between patients treated with DC-CCLA and patients treated with SARPE. This implies that a certain number of gingival recessions cannot be avoided with either treatment modality. Gender was found to have a significant effect on the incidence of gingival recession, with males being more affected (OR: 2.02), regardless of the type of intervention. Considering the unbalanced gender distribution and the small effect size found, the gender difference in the incidence of gingival recession should be interpreted with caution. The results indicate that the incidence of gingival recession was independent of the type of expansion appliance used. This is consistent with a recent systematic review showing that there were no statistically significant differences in skeletal and dental effects with bone-borne or tooth-borne SARPE [5].

It must be noted that multiple gingival recessions were already present in the adult population before treatment (T0). These findings align with the thesis that gingival recession is part of normal dental aging [19, 23]. Recessions were unevenly distributed within the dental arches, with some teeth more frequently affected. Differences in the prevalence of specific teeth compared to other studies, which found the highest prevalence in mandibular incisors, may be due to age differences or behavioral influences [17]. Post-treatment (T1), there was a significant increase in the prevalence of gingival recessions in both groups, indicating that both non-surgical and surgical crossbite correction in adults can exacerbate gingival recession. Regarding individual teeth, the distribution of gingival recessions within the arches was comparable to T0.

The incidence of gingival recession after crossbite correction (T1-T0) in our study was 6–8% at tooth level. It should be emphasized that these values are comparable to results from other studies that have investigated gingival recession with orthodontic treatment in general and not in adult crossbite correction in particular. According to our results, gingival recession with crossbite correction in adults was not more frequent than gingival recession with orthodontic treatment in general, and discrepancies in the incidence may be due to different age groups of patients, measurement sites, or evaluation methods [23, 24]. Surprisingly, there were also teeth where an existing gingival recession disappeared after treatment. We believe two factors may contribute to this observation. First, the presence of gingival hyperplasia - induced by the treatment with fixed appliances - could potentially influence the results as gingival hyperplasia could lead to a coverage of previously exposed root surfaces. Nevertheless, this applies more to the surgical group, as in the non-surgical group the brackets were bonded to the lingual surface, but buccal buttons for intermaxillary elastics were also used. Second, a torque movement of the root towards the center of the alveolar process seems to have a positive impact on the incidence of gingival recessions. We assume that some teeth did react favorably when the initial root prominence was reduced. It was shown that orthodontic torque correction can reduce gingival recession depth [28, 38] in teeth that were affected by the wire syndrome [39]. This implies that orthodontic treatment is not only a risk factor for gingival recession but orthodontic torque correction can also reduce gingival recession. Contrary, untreated buccal gingival recession depth in patients with good oral hygiene highly likely tends to increase during long-term follow-up [27].

**Fig. 6:**
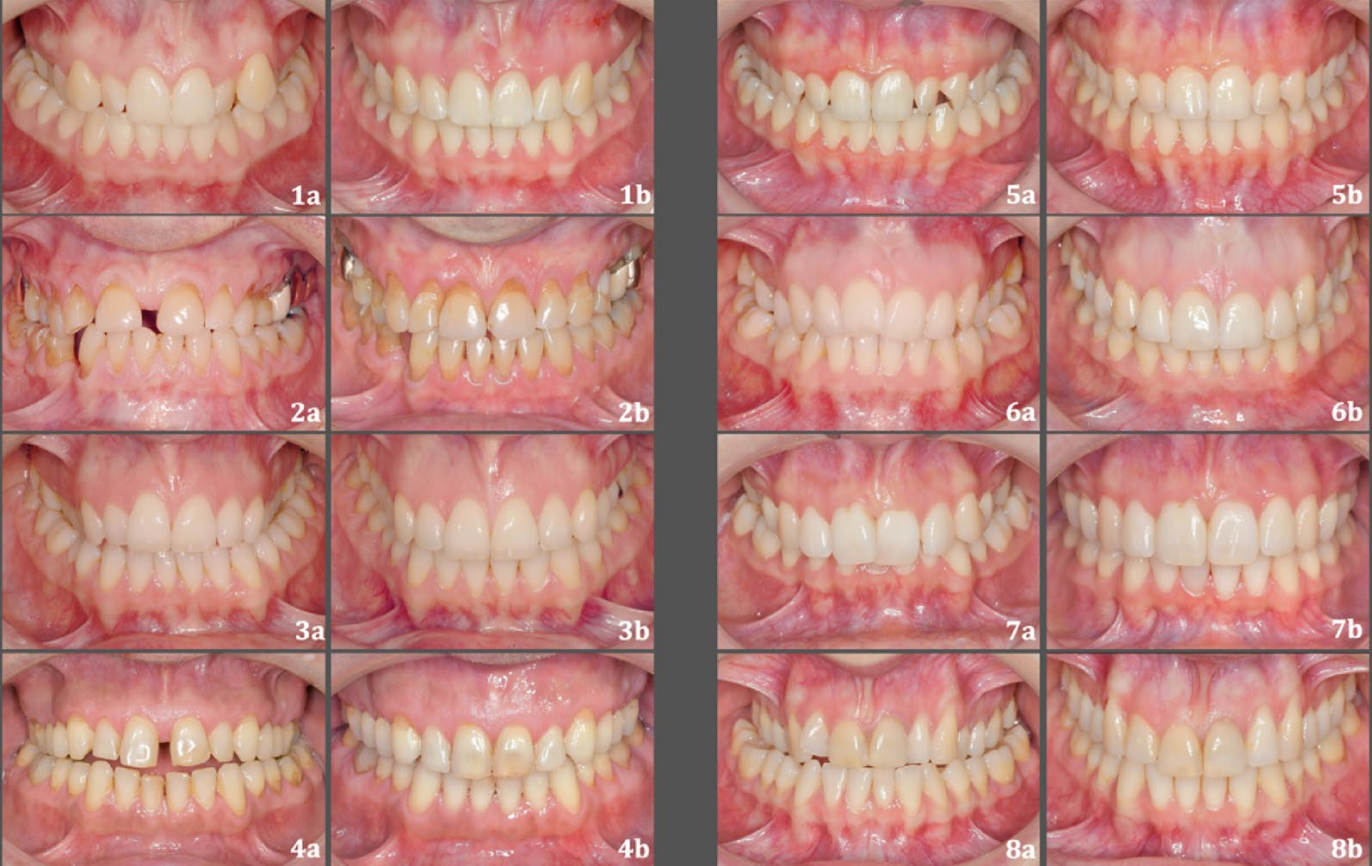
Eight example cases in the DC-CCLA group. **a** Pre-treatment situation. **b** Post-treatment situation after non-surgical crossbite correction with dentoalveolar expansion in the upper and dentoalveolar compression in the lower arch

**Fig. 7:**
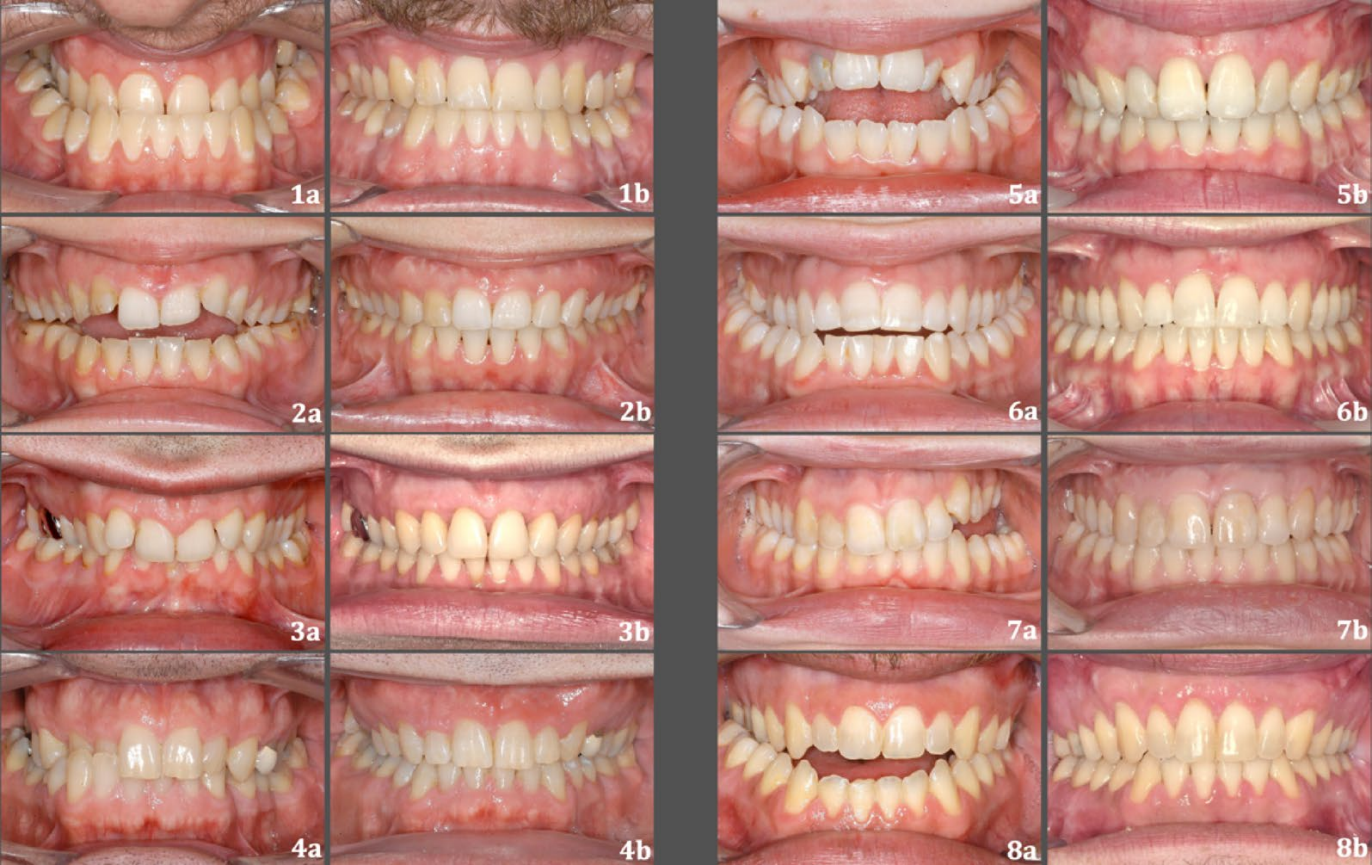
Eight example cases in the SARPE group. **a** Pre-treatment situation. **b** Post-treatment situation after surgical crossbite correction

There was a difference in the location of gingival recessions (T1– T0) between the groups, however, this difference was not statistically significant. In the SARPE group, when the corresponding teeth of both sides were counted together, maxillary central incisors were most often affected by gingival recessions, and the maxillary/mandibular second molars as well as mandibular canines were the least commonly affected teeth. It is evident that gingival recession is a complication associated with SARPE [12, 40]. Williams et al. [40] also found that gingival recessions with SARPE were most common at the central incisors. In the DC-CCLA group, when the corresponding teeth of both sides were counted together, maxillary first molars were the most often affected, and the maxillary central incisors were the least frequently affected teeth. It is assumed that gingival recession is associated with the amount of tooth movement. In the SAPRE group, a large diastema develops between the central incisors and large tooth movements were performed for diastema closure, which seems to increase the risk of gingival recession. In the DC-CCLA group, large tooth movements were performed for dentoalveolar expansion and compression in the posterior region, which also seems to increase the risk of gingival recession in this area. Figure 6 shows eight example cases in the DC-CCLA group and Fig. 7 shows eight example cases in the SARPE group. It appears that gingival recessions in the posterior region, that are less visible, may be preferred by our patients from an esthetic point of view. Similar to the results of other studies, patient age was a risk factor for the development of gingival recession [19, 41]. This suggests that the risk of developing gingival recession with crossbite correction in adults increases with age. Other studies have investigated the incidence of gingival recession with SARPE. The results indicate that the incidence is 8–29% [40, 42, 43] at patient level. Carmen et al. [42] found a 5.1% incidence of gingival recessions in the maxilla with SARPE at tooth level, which is consistent with our results of 5.8%. However, the development of periodontal trauma after orthognathic surgery is still controversial [44–46] and could be influenced by the type of incision to respect vascularization [47]. In our study, most of the patients in the SARPE group received a second Le Fort I osteotomy after SARPE and were therefore exposed to a marginal incision and flap in the maxillary anterior region twice. Bohner et al. showed that a marginal incision for Le-Fort I osteotomy did reduce papilla height one month after surgery but did not result in clinically significant changes in periodontal tissues and esthetics of the anterior zone six months after surgery [31]. In our study, gingival recessions were recorded after debonding, which was at least 6 months after the last surgical intervention in the SARPE group, and therefore the influence of the marginal incision seems to be limited. Time point T1 was selected in the SARPE group after removal of the fixed appliances to enable a direct comparison with the DC-CCLA group.

Regarding non-surgical crossbite correction in adults, it is often claimed that posterior teeth in the maxilla are displaced buccally through the alveolus which could lead to side effects such as gingival recession, bone loss and root resorption [48]. However, data on the development of gingival recession is limited and authors often describe their opinion or unpublished data [49, 50]. Handelman et al. investigated gingival recession in adults undergoing non-surgical rapid maxillary expansion. They found an incidence of new gingival recessions of 2.3%, which is slightly lower compared to our results [48, 51]. Discrepancies in incidence may be due to different measurement sites and evaluation methods. The authors state that the posterior dentition seemed to be translated with the alveolus rather than through the alveolus [48].

In orthodontics, the cortical bone is often considered as the limit for tooth movements [52]. However, we do not know the size of the bony envelope. Our previous study showed that translational tooth movements with transversal dentoalveolar compensation using CCLAs are possible [14]. We call this the “transversal Herbst effect”. Tipping movements lead to compressive forces in the occlusal and apical third of the root and could therefore cause alveolar bone loss due to the high strains acting on the root [53, 54]. In theory, bodily tooth movements could reduce these side effects in the transverse dimension. Capps et al. showed that buccal bone apposition is possible in buccal bodily tooth movements [55]. Bodily tooth movements can be achieved with CCLAs, for example, as these appliances offer excellent torque control due to the high precision of the slot–archwire combination [56–58]. Due to anatomical and iatrogenic factors, dehiscence and fenestration can occur during orthodontic treatment [59, 60]. Whether dehiscences necessarily lead to gingival recession remains controversial [26, 61]. It is important to mention that the detection of fenestrations and dehiscences using cone beam computed tomography (CBCT) is difficult and there is a risk of overestimation [62]. The reason for this is the spatial resolution. In clinical application, it seems difficult to assess bony structures smaller than 0.5 mm [63, 64].

### Strengths and limitations

As the study population did not change from previous studies [10, 14], similar limitations can be assumed. To reduce the risk of selection bias, consecutively treated cases were included, and no patient was excluded from the analysis for any reason other than the defined exclusion criteria. Patients in this study were treated in two orthodontic centers in Germany. The results may therefore not be fully generalizable to other orthodontic settings. The decision to use tooth-borne or bone-borne appliances was made on a case-by-case basis, contributing to the heterogeneity of the SARPE group. This must be considered as another limitation of the present study. The study design might limit the ability to detect subtle differences in gingival recession between bone-borne and tooth-borne SARPE. However, studies have failed to show significant differences for skeletal/dental effects and relapse with bone-borne or tooth-borne SARPE [5–8], and the results of the regression analysis indicate that the incidence of gingival recession was independent of the type of expansion appliance used. In this study, two treatment approaches are compared. Therefore, the influence of buccal versus lingual fixed appliances on the incidence of gingival recession can not be answered with this study design.

The main limitation of the present study is the point in time at which the data was collected. Gingival recessions were recorded after removal of the fixed appliances. It must be assumed that the incidence of gingival recession will increase significantly in the post-treatment period [23, 24]. We do not know if there will be a difference between the two treatment groups, but patients will be followed up to fully understand the long-term effects on gingival recession. However, the development of gingival recession may later regress to the mean [15]. The present study focuses primarily on the number of gingival recessions without considering the functional and aesthetic impact on the patients. It seems important to include patient perceptions in future studies. Future studies should also include multiple evaluations after debonding to investigate the effects of gingival hyperplasia and inflammation, ensuring that assessments of gingival recessions reflect true tissue changes.

The design of this study did not allow conclusions to be drawn about fenestrations or stability with both treatment modalities. To investigate fenestrations, CBCT would be required, which was not feasible for medical and ethical reasons. Furthermore, it remains questionable whether fenestrations can be detected with CBCT [64] and what the clinical implications are. A long-term longitudinal study is required to clarify the open questions about stability. The evaluation of digital models only and the lack of assessment of periodontal parameters such as gingival phenotype must be considered another limitation when interpreting the results. As a consequence, these results should be interpreted with caution. Future studies should include the gingival phenotype. However, the consensus-based approach for the registration of recessions aimed to increase the reliability and validity of the findings by reducing individual bias and promoting agreement between raters. A high intrarater reliability (κ = 0.876) reinforced the robustness of the data collection method. Finally, similar to the methodology of Renkema et al. [23], no statement can be made about the severity of the recessions and only labial gingival recessions were scored, which must be considered as another limitation of this study. However, it was shown that buccal recessions occur more frequently than lingual recessions [15].

There was a significant difference in Angle Class distribution - specifically a higher number of Class I cases in the DC-CCLA group, which could influence the incidence of gingival recession. This was expected as the SARPE group included surgical sagittal correction in all cases. However, regression analysis showed that the incidence of gingival recession was independent of the Angle Class. Vertical discrepancies have not been included in the analysis, which can be seen as another limitation of the current study. Open bite may theoretically contribute to altered lingual posture, but there is a lack of evidence for a direct association between altered tongue position and gingival recession. Lastly, the difference in observation time between the groups may impact the incidence of gingival recession, as longer treatment times may be associated with a higher risk of complications. However, the results of the regression analysis indicate that the incidence of gingival recession in our study was independent of the observation time.

## Conclusion

Within the limitations of this study, dentoalveolar compensation with CCLAs did not cause a significantly higher number of gingival recessions than SARPE after debonding.

## Data Availability

The datasets used and/or analysed during the current study are available from the corresponding author on reasonable request.
